# Editorial: Holistic approaches to understanding obesity and metabolic diseases in urban environments

**DOI:** 10.3389/fnut.2025.1737637

**Published:** 2025-11-21

**Authors:** Emmanuel Cohen, Mor Diaw

**Affiliations:** 1 IRL3189 Environment, Health, Societies, Dakar, Senegal; 2 UMR7206 Eco-anthropologie, Paris, France

**Keywords:** obesity, cardiometabolic health, determinants, holistic approach, urban transition

Although obesity has a complex multi-factorial etiology, requiring interdisciplinary expertise to discern the potential patterns of drivers involved in its increasing prevalence worldwide, most studies use a uni-disciplinary approach: psychological, sociocultural, physiological or genetics, a scientific strategy probably responsible for the current failure to prevent obesity, whose prevalence has tripled since 1975 according to WHO (1). Hence, the biocultural background of populations is insufficiently considered to assess their particular adaptive capacity for evolving in new urban environments, in order to minimize their exposure to the risks of obesity related cardiometabolic diseases (ORCDs). Although the generic determinants of obesity are well-understood, their respective effect as well as systemic interactions on BW regulation remain poorly understood. Moreover, the pathways predicting ORCDs between sociodemographic indirect determinants (age, sex, education, migration, etc.) and socio-ecological direct determinants (body weight norms, dietary intake and physical activity) are also rarely explored to study the etiology of ORCDs (2). Yet, both respective and cumulative contributions of these drivers, as well as the multiple driver pathways leading to ORCDs, must be explored to explain the difference in the prevalence and incidence of ORCDs between countries, and better identify at-risk subgroups toward this rising burden. The aim of this Research Topic consists in refocusing the study of obesity at an anthropological scale, by considering holistically populations' characteristics (sociodemographic and socio-ecological) that influence their degree of adaptation to obesogenic urban environments, to provide new insights to develop anthropologically relevant targeted programs to prevent and treat obesity and ORCDs.

Based on 13 relevant contributions, from an articulation between 11 original researches conducted worldwide, including 7 quantitative, 1 real-world, 1 mixed-methods and 2 longitudinal studies, and 2 reviews, this Research Topic, covering all age ranges, provided relevant findings as well as critical analyses and interpretations to deeply explore the synergetic effects of ORCDs' determinants in a globalized obesogenic urban environment. Through contributions at both national and regional scales, focusing on the complex etiology of obesity, ORCDs, as well as body composition and body fat distribution, covering all continents, the Research Topic: “*Holistic Approaches to Understanding Obesity and Metabolic Diseases in Urban Environments*” allows an original overview of factors leading to cardiometabolic diseases in countries situated at contrasted stages of the nutrition transition (1). This Research Topic has a focus on Low- and Middle-income Countries (LMICs) where the urbanization rates are the highest (3), as the incidence ORCDs (4). Consequently, this collective work will provide original insights to support innovative intervention strategies and programs aiming to reduce at mid- or long-term the ORCDs pandemic, with a variety of tools and skills to adapt them to local and population specificities (5).

Hence, two first contributions, from Jiao et al. and Zhang et al., explored, respectively, in Northwest and Southwestern China the determinants of ORCDs in adults. These quantitative cross-sectional studies showed that the urban transition occurring in both regions overexposed at-risk subgroups, i.e., women and middle-aged people, to ORCDs. This seems particularly true in non- or ex-smokers as well as in rural people experiencing a rapid shift in their lifestyle (e.g., increasing sedentary behaviors and energy-dense food), attesting that China presents an advanced stage of nutrition transition. The next contribution carried out by Szemik et al. focused longitudinally on the determinants overweight/obesity in another at-risk subgroups: the students. Indeed, this study implemented in an urban Polish area showed that the exposure to overweight/obesity increased across the years through specific risk factors as male sex, social insecurity and abundant meat consumption. Then, a real-world cross-sectional study conducted by Liu et al. in American Adults from the National Health and Nutrition Examination Survey demonstrated a negative association between the dietary intake of live microbes and obesity, mediated partially by the serum vitamin D. This suggests that foods with higher microbial concentrations could be beneficial for cardiometabolic health and vice versa. Another quantitative cross-sectional study, also carried out in the same population by Li et al., showed significant associations between three abdominal fat distribution indices—lipid accumulation product (LAP), body roundness index (BRI), and waist triglyceride index (WTI)—and osteoarthritis. In addition, stronger associations appeared in participants with lower education, hypertension (for WTI), without hyperlipidemia (for LAP) and non-smokers (for BRI). A literature review, conducted by Ard et al. in the United States, highlighted the evidence that a holistic integrated approach to obesity management, including campaigns fighting stigma, messaging encouraging the public to consider all levels of intervention, policy advocacy for anti-obesity medication coverage, and environmental change, results in better outcomes for people with obesity as it is a chronic, complex, and often relapsing disease.

Other contributions focused on the determinants of ORCDs in disadvantaged populations overexposed to obesity and/or barriers for health facilities access. A first contribution conducted in Hispanic American children by Vazquez et al. explored longitudinally how neighborhood opportunities, via a Childhood Opportunity Index (COI) illustrating the cumulative positive and negative environmental aspects (e.g., parent BMI and education, family income) involved in child health, are related to childhood weight status. Globally, all the higher COI levels were linked to healthier weight status compared to the lowest COI level. Then, a systematic review conducted by Jalilzadeh and Goharinezhad found worldwide 82 factors influencing overweight and obesity, categorized into five major groups: sociocultural, economic, technological, environmental, and political. Consequently, multi-sectoral interventions are required by the authors to address the obesity pandemic, especially in vulnerable population groups mainly concentrated in LMICs where studies on public health nutrition remain scarce. Another contribution conducted by Chagas et al. focused quantitatively, through a cross-sectional study, on the determinants of body composition among adult outpatients of a public university hospital specializing in cardiology in Northeastern Brazil. Distinct factors were found to influence visceral and subcutaneous obesity (VAT and SAT): sedentary behavior emerged as an independent predictor of visceral obesity while alcohol consumption was associated with a subcutaneous obesity pattern. In addition, diabetes and sociodemographic factors (older age, non-White race, and lower education) were predictive of an elevated VAT/SAT ratio.

Kawalkar et al. found in their mixed-methods study conducted among adult patients with type 2 diabetes mellitus (T2DM) of the tertiary care hospital in Vidarbha region of India that male participants exhibited higher awareness levels across various aspects of dietary recommendations. Because female participants presented lower knowledge in health related nutrition including recommended fruit and vegetable intake, milk product consumption, and types of fruits suitable for diabetics, the conclusions consequently emphasize gender-sensitive educational interventions to enhance diabetes-related nutrition knowledge and self-management practices among T2DM patients. Then, another cross-sectional quantitative study conducted by Ebrahimi et al. among adult Iranian women with overweight and obesity, who were referred to health centers of the Tehran University of Medical Sciences, observed that participants with higher lifestyle risk score, including SES, education levels, sleep quality, waist-to-height ratio, dietary behavior (vegetables, grains and legumes intake) and physical activity, had higher body mass index associated with metabolically morbidities. The authors recommended further prospective studies to advance understanding of the relationship between lifestyle and obesity in the country.

Yimer et al. carried out a cross-sectional quantitative study among urban bank employees from Ethiopia (Dessie) to explore the determinants of abdominal obesity. As expected, the mean waist circumference among this group was high, with more than half having central obesity. Being older and married, consuming discretionary calorie, and presenting lower metabolic equivalence task hours (as a proxy of physical activity) were the significant factors of waist circumference. From these interesting findings, the authors suggested a promotion of healthy activities such as leisure-time physical activity and lower discretionary calorie intake in this at-risk subgroup. The last contribution in the Democratic Republic of Congo (DRC), conducted by Wakilongo et al., was a rural-urban cross-sectional study among adult women focusing quantitatively on the determinants of overweight and obesity. A binomial logistic regressions highlighted that overweight-obesity has a significant positive correlation with: duration of urban residence, socio-economic status (SES) and valorisation of stoutness. In addition, a pathway analysis based on a structural equation model, showed that urban residence and SES were associated with an increase in overweight-obesity, with a positive correlation with processed food consumption and a negative correlation with physical activity. Age was associated with an increase in overweight-obesity through a negative association with physical activity, whereas stoutness valorisation directly increased overweight-obesity. The conclusions advised that the DRC public authorities should consider how socio-demographic and socio-ecological factors contribute jointly to the rising prevalence of overweight-obesity in the country.

These fruitful, diverse and complementary contributions made this Research Topic: “*Holistic Approaches to Understanding Obesity and Metabolic Diseases in Urban Environments*” innovative and relevant to explore worldwide, through various indicators, overweight, obesity, ORCDs and its determinants. Health nutrition related behaviors (e.g., sedentary behaviors and energy dense food) were pointed out as major factors of the obesity pandemic within the globalized urban transition. Several at-risk subgroups were also identified to be overexposed to this public health issue, especially elders, women and ethnic minorities. The Research Topic also highlighted the necessity to concentrate the efforts on LMICs where the incidence rates of obesity are the highest while public health infrastructures to deal with this phenomenon are often insufficient. The critical aspect to be considered in LMICs is the promotion and the development of nutritional education programs to prevent and/or reduce the rising obesity pandemic, while the social exclusion of minorities in high income countries must be addressed to fight against this persisting burden in this region of the world. In all socio-ecological contexts, the intervention programs shall adopt a holistic approach of the determinants of obesity and ORCDs to obtain better results for their prevention and reduction.
